# Structure from Motion Multisource Application for Landslide Characterization and Monitoring: The Champlas du Col Case Study, Sestriere, North-Western Italy

**DOI:** 10.3390/s19102364

**Published:** 2019-05-22

**Authors:** Martina Cignetti, Danilo Godone, Aleksandra Wrzesniak, Daniele Giordan

**Affiliations:** 1 National Research Council, Research Institute for Geo-Hydrological Protection, 10135 Turin, Italy; martina.cignetti@irpi.cnr.it (M.C.); aleksandra.wrzesniak@irpi.cnr.it (A.W.); daniele.giordan@irpi.cnr.it (D.G.); 2 Department of Earth Sciences, University of Pavia, 27100 Pavia, Italy

**Keywords:** unmanned aerial vehicle (UAV), structure from motion, landslide-infrastructure interaction, photogrammetry, deep-seated gravitational slope deformation

## Abstract

Structure from Motion (SfM) is a powerful tool to provide 3D point clouds from a sequence of images taken from different remote sensing technologies. The use of this approach for processing images captured from both Remotely Piloted Aerial Vehicles (RPAS), historical aerial photograms, and smartphones, constitutes a valuable solution for the identification and characterization of active landslides. We applied SfM to process all the acquired and available images for the study of the Champlas du Col landslide, a complex slope instability reactivated in spring 2018 in the Piemonte Region (north-western Italy). This last reactivation of the slide, principally due to snow melting at the end of the winter season, interrupted the main road used to reach Sestriere, one of the most famous ski resorts in north-western Italy. We tested how SfM can be applied to process high-resolution multisource datasets by processing: (i) historical aerial photograms collected from five diverse regional flights, (ii) RGB and multi-spectral images acquired by two RPAS, taken in different moments, and (iii) terrestrial sequences of the most representative kinematic elements due to the evolution of the landslide. In addition, we obtained an overall framework of the historical development of the area of interest, and distinguished several generations of landslides. Moreover, an in-depth geomorphological characterization of the Champlas du Col landslide reactivation was done, by testing a cost-effective and rapid methodology based on SfM principles, which is easily repeatable to characterize and investigate active landslides.

## 1. Introduction

Mountain regions have a remarkable exposure to landslide hazards, which are considered one of the most widespread natural disaster throughout the world [1,2]. In particular, in alpine regions, landslide types range from localized and sudden rock falls, to widespread and progressive rotational or planar slides, as well as large complex and in-deep landslides [3,4,5]. The occurrence of landslides of diverse types and size, and their local activation or reactivations play an important role in mountainous landscape evolution, often causing significant damage and casualties [6,7].

Active landslide inspection and monitoring are key elements in the assessment of landslide behaviour, specifically to support landslide emergency management and to identify areas with the highest damage. Usually, accurate landslide hazard evaluation needs consistent data concerning the extent of the unstable slope, detection of morphological features, deformation dynamics, triggers and historical records. This information is also useful to define landslide hazards and vulnerable areas [8,9,10]. Accurate landslide damage assessment and mapping requires time-consuming and costly methodologies by expert professionals (e.g., geologist, engineers) and field campaigns.

Remote sensing technologies, e.g., photogrammetry, terrestrial and airborne Light Detection and Ranging (LiDAR) are renowned techniques for landslide analysis [11,12]. The obtained orthoimages and Digital Terrain Models (DTMs) supply detailed representations of the topographic surface, which provide tools for recognizing landslide types and identifying related surface morphology signatures [12,13,14,15]. Aerial photogrammetry constitutes a suitable approach to obtain medium to high resolution datasets, enabling the definition of the geometric characteristics of the observed phenomenon [16] and multi-temporal analysis of the slope evolution [17,18,19]. Also, the LiDAR survey provides a useful instrument to study natural hazards through its high-resolution DTMs analysis [20,21,22]. However, these lengthy, in terms of data acquisition and processing, and costly procedures might not be suitable during an emergency when a more rapid overview of the current state is necessary. Therefore, straightforward and repeatable procedures that are able to provide reliable datasets in a very short time and in a safe manner are necessary.

Nowadays, the spread of unmanned aerial vehicles (UAVs) in natural hazards assessment and characterization has significantly increased [23,24,25,26,27], especially when there is a need to rapidly obtain very high-resolution airborne imagery of the area of interest. Recently, the use of UAV or Remotely Piloted Aircraft System (RPAS) has become more commonplace thanks to the improvement of autopilot or semi-autopilot systems, high-resolution digital cameras, and GNSS and inertial systems.

Captured images are geocoded by various approaches requiring on board and/or ground based approaches. In fact, UAVs are usually equipped with positioning apparatuses. In order to improve positioning accuracy, ground control points (GCPs) are positioned, surveyed and used in image processing. Additionally, on board GNSS can be coupled with ground receivers to measure the UAV position with an RTK approach [28,29]. Geocoded images are then used to generate a 3D point cloud, and consequently, a Digital Elevation Model (DEM) and orthoimage. These map layers are suitable for different mapping and analysis purposes by manual or automatic procedures that exploit image features (e.g., colours, texture) or DEM’s geometrical characteristics (e.g., slope, height differences) by applying, respectively, image segmentation, classification or morphometry [30,31]. Obtained products are usually validated by comparison with previous datasets as LiDAR data or GNSS surveys in order to quantify the reliability of the procedure [32]. It is important to note that the employment of UAV must, in addition to technical requirements, meet local regulations in order to assure the security of the operation [33].

A well-established practice is observed in engineering geology, especially in landslide mapping [34,35,36], in slope stability analysis [23,37], as well as in a wide variety of natural hazards investigation, such as fluvial [38], volcanic [39] and glacial [40]. Moreover, UAVs are frequently employed during an emergency in order to provide information on structural damage and to define a preliminary impact assessment [41,42].

Focusing on the study of landslide hazards, the employment of UAVs is a useful solution for acquiring images at sub-decimetric resolution, especially for small active phenomena. They can be employed for a 3D model reconstruction by applying the Structure from Motion technique, and Multi-View Stereo (MVS) algorithms [43], a relatively new image processing technique based on computer vision algorithms, which obtain very high-resolution Digital Elevation Models and orthoimages.

The UAV survey, an accurate and cost-effective technique with a more streamlined process compared to the classic approaches such as aerial photogrammetry and field survey, provides high spatial and temporal resolution photographs of the area of interest [24]. The visual interpretation of orthoimages allows the implementation of landslide inventory and geomorphological characterization of the investigated sites [44]. With a multi-temporal analysis, the orthoimages time series provide the surficial of the unstable area, which is computed by the comparison of well recognizable features [45,46,47]. Instead, the DEMs time series provides detailed multi-temporal sets of 3D surfaces that are useful for vertical displacements investigation [48,49]. Moreover, the very high-resolution of these models allows the detection and mapping of all the geomorphological features due to landslide evolution [44,47].

Recent technological improvements allow the customization of UAV by equipping them with different cameras in order to acquire both RGB images and also different bands to be used in indices computation for the identification of anomalies in water content [50], and vegetation health anomalies [51].

In this paper, an image-processing workflow based on the use of Structure from Motion algorithm applied to UAVs datasets, old aerial images, and terrestrial photos have been implemented for a detailed characterization and monitoring of active landslides. The presented methodology has been developed and applied to the Champlas du Col landslide, Sestriere municipality, Piemonte Region, north-western Italy. Thanks to the availability of different datasets, we tried to achieve the following goals: (i) generation of a landslide inventory of the area of interest through aerial photogram interpretation and historical mapping of landslide activities; (ii) definition of the landslide boundary, (iii) geomorphological characterization of the investigated phenomenon, principally by exploiting RPAS products, and (iv) semi-quantitative analysis of the displacement that occurred between the two RGB acquisitions and by terrestrial photos processing.

The purpose of the proposed approach is to provide a rapid and low-cost solution for a multi-temporal and multi-scale analysis by exploiting multi-source datasets. It should provide a detailed geomorphological map of an active landslide and a temporal and spatial investigation of its behaviour, aimed to provide situational awareness immediately after the emergency phase and supply information for crisis management.

## 2. Study Area

**Figure 1:**
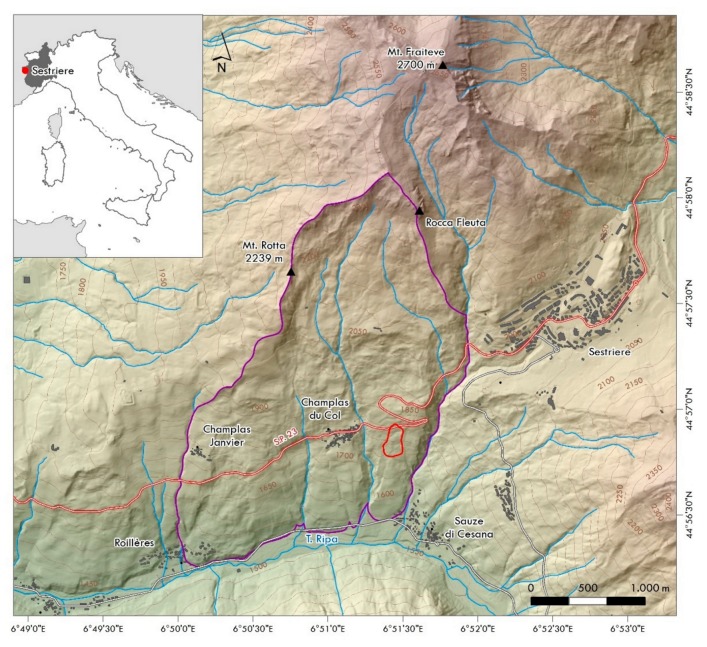
Geographic location of the area of interest, close to the Champlas du Col hamlet, Sestriere municipality (Piemonte, north-western Italy), red polygon corresponds to the May 2018 Champlas du Col landslide reactivation, while the purple one corresponds to the Champlas du Col deep-seated gravitational slope deformation (DsGSD).

The area of interest is located at the confluence of the Upper Susa Valley and the Chisone Valley (northern Cottian Alps), between the localities of Sauze di Cesana and Rollières, Sestriere municipality (Piemonte Region, north-western Italy). The study was carried out on the Champlas du Col landslide, located on the south-facing slope of Mt. Fraiteve (2700 m a.s.l.), close to the namesake hamlet (Figure 1).

The Champlas du Col landslide is a complex movement with a source area affected by a rotational component in the upper sector, which downhill, turns into an earth flow. The landslide extends between elevations of 1810 m at the crown, which involves the S.P. 23 national road, the principal way to reach the town of Sestriere, one of the most famous ski resorts in the north-western Italian Alps, and 1680 m, with an average slope of about 23°.

**Figure 2:**
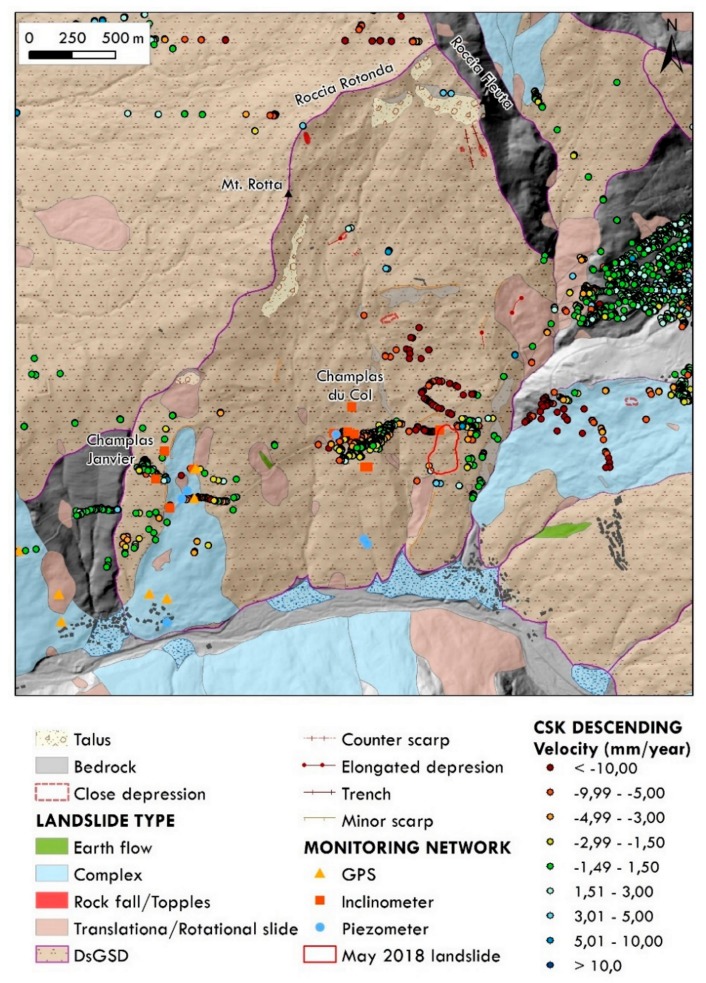
Geomorphological map of the Champlas du Col Deep-seated Gravitational Slope Deformation, landslides associated (landslide polygons source: SiFraP, Arpa Piemonte web-site [62]), in situ instruments (data source Arpa Piemonte web-site [62]) and remote sensing Cosmo-SkyMed (CSK) SAR data distribution (data source: Portale Cartografico Nazionale [63]); red polygon corresponds to the May 2018 reactivation and the purple one to the Champlas du Col DsGSD.

At the slope scale, the Champlas du Col landslide is embedded in an extensive instability phenomenon that corresponds to a Deep-seated Gravitational Slope Deformation (DsGSD) [5]. This DsGSD involves the majority of the valley south-facing flank of Mt. Fraiteve (515.15 ha), ranging from 2488 m, between the two peaks of Roccia Fleuta (2456 m) and Roccia Rotonda (2402 m), and down toto the bottom of the main valley. This huge phenomenon involves the metasedimentary successions of the Cerogne-Centiplagna unit, mainly constituted by heterogeneous calcschists. According to [52], the DsGSD movement corresponds to creep processes along discrete sliding surfaces, and along multiple listric surfaces. The movement rate, derived from high-resolution SAR images processing (i.e., COSMO-SkyMed (CSK) dataset from the WMS service of the *GeoPortale Piemonte* [53]) from 2011 to 2014, ranges from 20 to 35 mm/year along the LOS direction, for the area along the S.P. 23 way right above the Champlas du Col landslide. It decreases in the sector between the hamlets of Champlas du Col and Champlas Janvier, ranging from 5 to 10 mm/year. Generally, the SAR data coverage is poor with Permanent Scatterers (PS_s_) distribution mainly located in urbanized areas and along the road network (Figure 2). No SAR data are available for the Champlas du Col landslide due to the phase decorrelation effect [54,55] caused by the large displacement of the slide. In addition, between Champlas Janvier and Champlas du Col hamlets, several geognostic surveys with in situ instruments (i.e., inclinometers, piezometers) were carried out and some GPS benchmarks have been installed (Figure 2).

The Champlas du Col landslide has been known since the beginning of last decade, with repeated reactivations that are principally related to snow melting in early spring. In the spring of 2017 (April–May), a noticeable reactivation was observed, with evident damage on the surface road (S.P. 23) and to the water regulation infrastructure. In this framework a GPS benchmark was installed by the Regional Environmental Protection Agency (Arpa Piemonte) at the base of the retaining wall of the S.P. 23. The movement rate, measured from the end of May 2017 to October 2017, corresponds to about 10 cm for the observed period. The last reactivation, recorded during late April–early May 2018, was more intense and severe if compared to the previous one, with a prevailing component of movement as earth flow. This movement seriously endangered the S.P. 23, causing the interruption of the national road with damage to the road surface (e.g., fractures, steps) and to the remedial facilities (e.g., upstream and downstream retaining walls). During the emergency phase, the Metropolitan City of Turin (MCT) (the owner of the street) performed an in situ measurement campaign by the employment of a Robotic Total Station (RTS). A total displacement, from May 2017 to May 2018, of about 1.8 m was recorded, of which 1.3 m was recorded in the 2018 reactivation, with a deformation rate of 5 cm/day (data derived from SiFraP [56] Champlas du Col landslide schedule).

The Champlas du Col landslide, on the basis of the present state of knowledge, mainly seems to involve Quaternary deposits, primarily represented by the glacial deposits of the “Supersintema of Moncenisio” [57], and the ophiolitic unit of the “Cerogne-Ciantiplagna unit” (Cretaceous Inf.) [57]. Glacial deposits consist of *Diamicton*, poorly sorted unconsolidated sediment with clasts and blocky suspended in an abundant silty, silty-sandy matrix. Instead, the Cerogne-Ciantiplagna unit is part of a complex system of tectonostratigraphic units of different paleogeographic origin, forming the Pennidic system, bordered and/or crossed by important post-metamorphic fault systems that are variably oriented [52]. This unit consists primarily of monotonous sequences of calcschists with Epidote and micaceous marble including lenses of serpentinites and serpentionischists.

Morphologically, tectonic features strongly influenced the drainage network and landscape morphology, as testified by the Dora Riparia and Chisone rivers and tributaries pattern, which is controlled by NE-SW and NNW-SSE fault systems. Moreover, glacial morphodynamics prevail and influenced the past and actual slope setting, principally related to the debuttressing caused by glaciers retreat, strictly related to the development of large slow-moving instability, i.e., DsGSD_s_ [58,59].

From a climatic point of view, the study area is characterized by a temperate cold climate [60] typical of Alpine areas with a low rainfall regime (600–900 mm/year), and two peaks in autumn and spring [61]. During winter seasons, abundant snowfalls are recurrent.

## 3. Materials and Methods—Structure from Motion Multisource Approach

In this paper, the low-cost photogrammetric Structure from Motion (SfM) method was employed to characterize and monitor an alpine landslide, starting with the integrated use of multisource images an acquisition approach, and also taking advantage of low-cost solutions.

### 3.1. Data Acquisition

**Figure 3:**
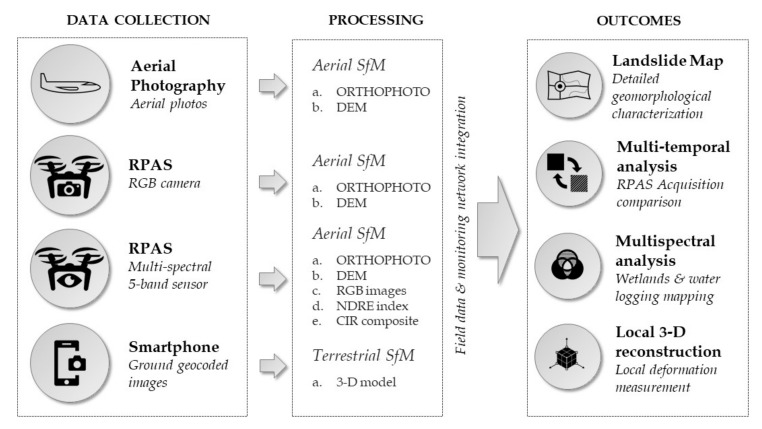
Flow chart of the Structure from Motion (SfM) multisource application methodology for landslide investigation.

Through a multiscale analysis, we developed a methodology that combines terrestrial and aerial image acquisitions (Figure 3). We generated a series of datasets composed of high-resolution images suitable for the SfM reconstruction process by using the Remotely Piloted Aerial System (RPAS), smartphone camera, and historical aerial photograms collected from diverse regional flights. The collected datasets are processed with Agisoft Photoscan, a commercial software that uses SfM to reconstruct the scene.

First, the aerial photograms derived from available aerial flights (IRPI internal picture library [64]) were collected. Five datasets from 1954 to 2008, with variable scales from 1:38.000 to 1:10.000 are shown in Table 1. Each dataset was than processed by SfM in order to obtain both orthoimages and digital elevation models (DEMs) of the area of interest. A set of markers (discernable points, e.g., a road crossing, a building) were selected in order to geocode the processed raster data. The obtained orthoimages and associated DEMs resolution ranged from 25 cm/pixel to 1.2 m/pixel.

**Figure 4:**
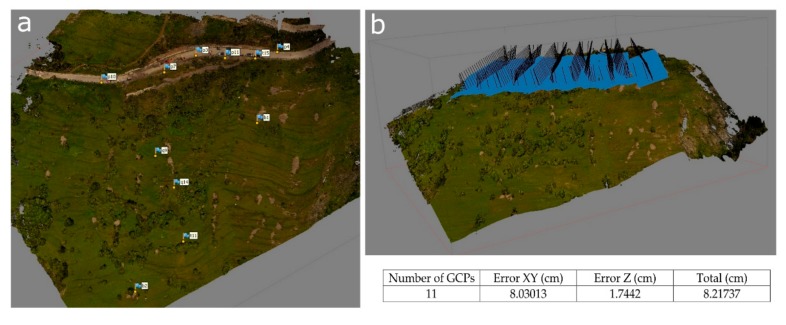
14 June 2018 UAV flight planning: (**a**) Ground control points (GCPs) distribution, with XY and Z errors associated, and (**b**) camera positions.

Upon request of the Civil Protection Agency of the Metropolitan City of Turin, the CNR-IRPI carried out two RPAS surveys, performed by two different quad-rotor UAV-systems (Table 2). On 15 May 2018, 81 aerial photos were taken by the UAV equipped with an RGB camera, flying in the most affected area across the S.P. 23. Flight planning was carried out on site (Figure 4) in order to pick a safe location for take-off and landing and define the area to be observed. The flight altitude was about 70 m to provide a ground resolution of approximately 5 cm per pixel. Before the UAV survey, 12 square reference targets (GCPs) were placed outside the unstable area for georeferencing purposes.

A second RPAS survey was planned on 14 June 2018 to acquire RGB and multispectral images. In this case, the UAV was also equipped with a 5-band (i.e., Blue, Green, Red, Red-Edge and near-IR) multispectral camera (REDEDGE [65]). In this case, 383 aerial photos were taken by the UAV-system flying over the whole unstable area. Eleven GCP_s_ were placed (Figure 4). The obtained orthoimage has a final resolution of 1.65 cm per pixel.

The GCP_s_ were surveyed using a Leica 1200 GNSS receiver in VRS-RTK mode (accuracy ±3 cm). The SfM approach, thanks to the GCPs collimation, allowed the generation of orthorectified and mosaicked images. The first identification of the landslide border was based on this first RPAS survey combined with field survey observations.

Prior to the flight, the sensor was calibrated with the panel provided with the camera, then the acquired images were processed with the same approach as the RGB ones in order to compute an orthomosaic for each acquired band as the Agisoft Photoscan software is capable of processing multiband images in a unique procedure.

Finally, we used the same processing approach for 38 images (2976 × 3968 pixels) taken manually with a 3.95 mm focal length camera. These pictures were taken in a local sector of the landslide along the lateral landslide limit. Nine benchmarks of 20 × 20 cm were placed within the scene to implement the SfM approach and make the 3D-reconstruction geometrically measurable.

### 3.2. SfM Data Processing

We used the generated dataset to identify slope landforms and define the landslide surface area through an analysis of morphological features and their variations, and a semi-quantitative analysis of the landslide deformation rate.

Taking advantage of the historical photograms (Table 1), we carried out a qualitative analysis of the landscape evolution over a 54-year period. Specifically, a landslide inventory of the whole area of interest was created by the interpretation of the multiple sets of aerial photographs, using stereo interpretation and directly digitalizing polygons in the GIS environment [66]. For the generation of this inventory, we used the geo-referenced orthoimages obtained by SfM processing. We analyzed each set of aerial photograms by identifying landslides in the 1954–2008 time span. We started by analyzing the 1954 dataset in order to identify the long-standing landslides. Then, by combining all the diverse sets of images, we analyzed all the subsequent landslides that occurred and the potential reactivation of the already identified phenomena. We distinguished the relative age of the identified phenomena, defining a landslide hierarchy by discriminating between the oldest phenomena, i.e., ancient landslides and the possible variation identifiable in the period between two subsequent flights. Landslides were also classified according to their type of movement, according to Cruden and Varnes [67] classification, and subsequently, Varnes [68] and Hungr [69], combining photogram analysis with the information retrieved from the public regional inventory of Piedmont, i.e., SiFraP [56]. After the identification of landslides based on the analysis of aerial images, we also used the regional digital terrain model, and the available information in the regional landslides inventory [56]. 

The availability of very high resolution RPAS imagery allowed a thorough depiction of the morphological features resulting from the May 2018 landslide reactivation and its extension. Moreover, the availability of two subsequent RPAS surveys allowed a multi-temporal analysis of the unstable area, to evaluate its changes.

Thanks to the possibility of equipping the UAV with a multi-objective camera featuring 5 band acquisition, multispectral analysis was done. In an open source GIS environment, diverse indices and RGB composites were computed by applying raster algebra tools, in order to detect anomalies in water content and vegetation health. As in previous works [50,70], we took advantage of the sensor features by computing the Normalized Difference Red Edge (NDRE) index, which uses the ratio of Near-Infrared and the edge of Red, and Color Infrared (CIR) composites [51] to identify water springs, wetlands and soil saturation within the unstable area. 

Moreover, three field surveys were carried out between May 2018 and March 2019 to detect the main geomorphological features of the active landslide. We also focused on and mapped the presence of wetlands and sources. The field data acquisition was supported by the use of “LocusMap”, an app dedicated to recording and collecting geocoded points and images.

During these surveys, we acquired different datasets along the main lateral. The SfM processing of these terrestrial images was used to create a 3D terrestrial model, and an expeditious evaluation of the displacement was recorded referring to a local sector of the unstable area by a low-cost solution. 

### 3.3. Additional Information

The obtained dataset has been improved using monitoring data available for this area. In particular, inclinometer and piezometer monitoring results were granted by the MCT. These instruments were installed by MCT after the 2018 spring reactivation. The inclinometer showed the presence of two shear surfaces at the depths of 3.5 m and 24.5 m. The availability of this data was very useful in the calibration of the 3D model of the slope and the definition of the landslide geometry.

## 4. Results

In this section, the results obtained by the application of the multisource and multiscale approach are presented as follows: (i) Landslide inventory map and multi-temporal analysis by aerial images acquired by aerial photography; (ii) Multi-temporal analysis acquired by RPAS; (iii) Multi-spectral analysis by RGB camera mounted on RPAS; (iv) Terrestrial images for 3-D reconstruction; and (v) Overall temporal evolution.

### 4.1. Landslide Inventory Map and Multi-Temporal Analysis by Aerial Images Acquired by Aerial Photography 

The 54-year aerial photography analysis allowed us to generate a landslide inventory of the entire study area. The observed landslides reveal a typical alpine landscape, characterized by a number of DsGSD_s_ that involve entire valley flanks, e.g., the Champlas du Col DsGSD (515.15 ha), which are associated to other subsequent phenomena, mainly represented by complex and translational/rotational landslides.

All the landslides, activated on the DsGSD_s_, were recognized by combining all the multiple sets of images. A landslide hierarchy was established that distinguishes four different generations on the basis of geomorphological criterion. The DsGSD_s_ bodies have been considered separately. The oldest landslides correspond to the first generation of landslides. These phenomena are generally large in size, exhibiting signs of severe reshaping from watercourse actions and from the ongoing slope instabilities over time. They partially preserve the typical morphologies of landslides (e.g., concave-convex shape, scarps, counter scarps) identifiable on each of the available flights. However, these landslides are partially obliterated by ancient agricultural practices and others human activities. Considering only the Champlas du Col DsGSD, all of the first-generation landslides covered an area of about 71.4 ha, prevalently involving the downstream portion of the DsGSD of Champlas du Col, sometimes crossing the road network and the Champlas Janvier hamlet. 

**Figure 5:**
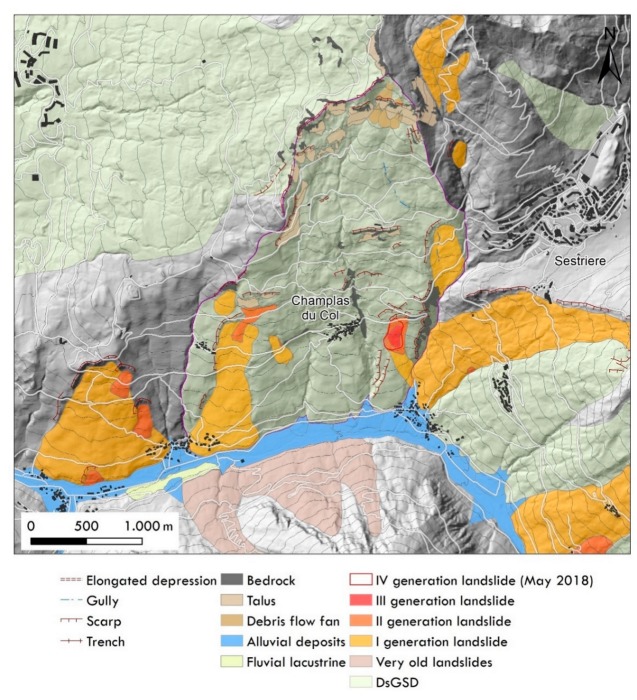
Landslide map of the area of interest distinguished on the basis of temporal hierarchy.

Figure 5 portrays the inventoried landslides spatial distribution on the whole area of interest, in association with some geomorphological features that are well recognizable and related with the evolution of the slope and prevalently due to landslides (e.g., main scarp, trenches, elongated depression). Within the first-generation landslides, we recognized two additional generations of landslides that have progressively altered or partially erased the morphology of these previous ones. The fourth generation is represented by the May 2018 landslide activation, who interrupted the main road.

**Figure 6:**
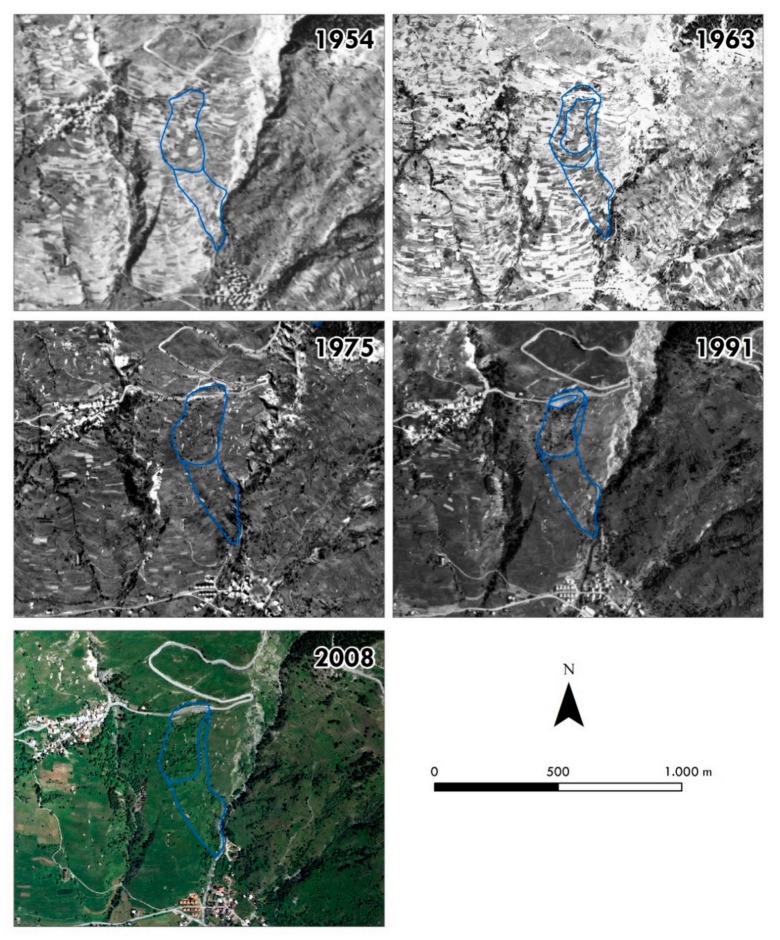
Orthoimages processed by SfM for the Champlas d Col area; the blue polygons represent the photo-interpreted Champlas du Col landslide bodies.

Focusing on the Champlas du Col sector (Figure 6), plotting the aerial photos allowed us to distinguish three chain-linked landslide bodies: (i) first-generation Champlas landslides; (ii) second-generation Champlas landslides; and (iii) third-generation Champlas landslides. The first and the second-generations bodies are already well recognizable in 1954 images, while the third-generation bodies were clearly detected in the 1963 aerial photos (Figure 6). Until 1963, the air photos clearly depict the complexity of the field pattern, which are mainly represented by agricultural land (i.e., pastures). In general, the agricultural parcels’ orientation is regular, following the contour lines, while in the Champlas du Col landslide bodies, an irregular and uneven orientation is clearly visible. From the 1975 orthoimage, we can note a general abandonment of land in terms of agricultural practice. The most prevalent land use is pasture featuring the absence of tree cover. Only in correspondence to the landslide sector, small bushes and sparse trees are present. It should be noted that the 1975–1991 inter-period variation of the second-generation Champlas landslide, highlighted by a new ‘fresh’ scarp set in the downstream portion of the S.P. 23 road, is clearly visible in the 1991 orthophotos.

### 4.2. Multi-Temporal Analysis Acquired by RPAS

The Champlas du Col landslide reactivation in May 2018 led to heavy damage, causing the closure of the national road (S.P.23). During the emergency, two UAV surveys were performed: one immediately after the reactivation, occurred at the beginning of May and the other after a month. The RPAS ensured fast processing, was able to provide highly accurate digital elevation models (DEMs) and provided very high resolution orthophotos of the Champlas du Col landslide. The orthophotos analysis, which was associated with the DEMs derivative products (e.g., shaded relief, slope) led to the mapping of all the geomorphological features of the slope due to the landslide and its evolution, and definition of the landslide boundaries. Furthermore, to exploit the multi-temporal acquisition, a semi-quantitative analysis of the potential changes that occurred between the two flights was computed.

#### 4.2.1. Champlas du Col Landslide Mapping with RPAS High-Resolution Images

The geomorphological evidence and the potential dynamics of the unstable area have been visually investigated by taking advantage of the two UAV imagery sets. The high-resolution RGB orthomosaics (5 cm resolution) supplied a detailed view of the surveyed area, and enabled us to map all the morphological features visible on the 15 May and 14 June orthoimages. In addition, the high-resolution DEMs supplied a detailed representation of the surface. The comparison of the DEMs derivative products such as shaded relief, slope, and contour with orthoimages allowed investigation and mapping of the morphological features that occurred due to the landslide evolution.

**Figure 7:**
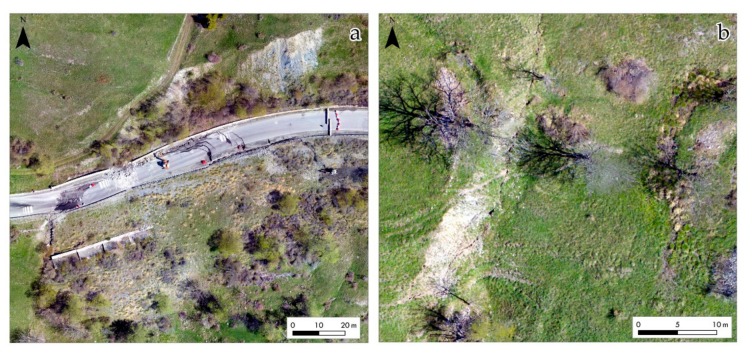
15 May 2018 UAV orthoimage detail: (**a**) Main scarp of the crown area and road damage; (**b**) clearly visible latera fracture associated with a local fresh scarp, in the middle portion of the right-limit of the unstable area. On the right of the picture one of the wetlands is vividly discernible.

The crown area is evidenced by an ENE-WSW trending scarp extended around a hundred meters in length (Figure 7a), and it ranges from 1800 m to 1820 m a.s.l. in elevation. In correspondence of this scarp, there are outcrops of the highly schistose calcschists of the Cerogne-Ciantiplagna unit. The zone of visible depletion involves the surface of the S.P.23 national road, causing noticeable fractures and steps on the pavement. In the downstream sector, a series of transverse fissures with variable extension (from 5 to 30 m) were recognized. Across the slope, starting from the downstream sector of the road, two main lateral fissures of around 100–150 m in length and a noticeable gap in the range of decimeters were observed (Figure 7b).

These evident fractures are associated to en-echelon tensional cracks, related to the drag of the slide. The right-side fracture involves the already in-place retaining wall, which shows clearly visible deformations. In the halfway sector, this main fracture is associated to a “fresh” scarp, that extends from 1750 m a.s.l., for about 20 m and about 3 m in height (Figure 7b). On the left-side, at about 1760 m a.s.l., two NNE-SSW trending minor scarps with variable extension of 30–40 m and an average height of a few meters were recognized. They are located in the proximity of the left-side lateral fracture.

**Figure 8:**
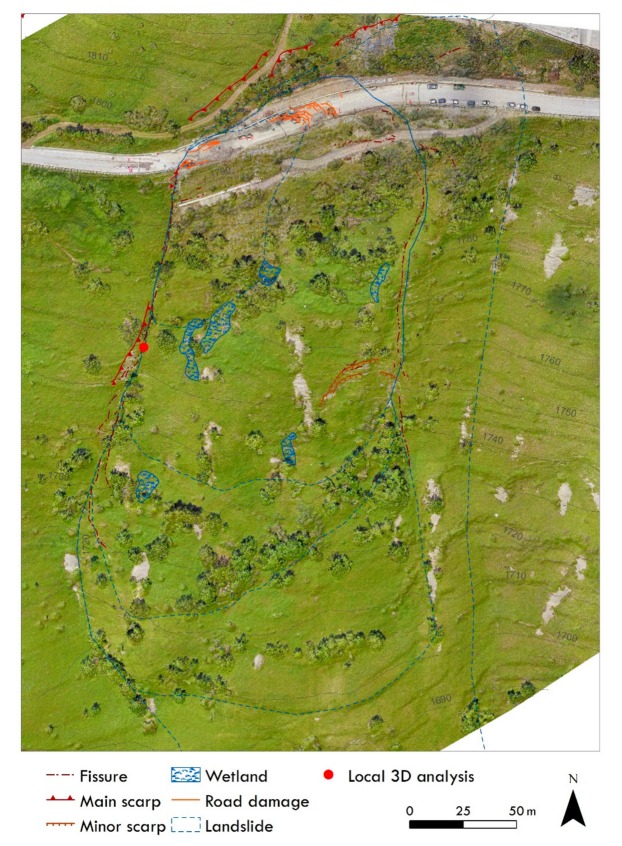
Map of the geomorphological evidence of the unstable area (Orthophoto source: 14 June 2018 UAV survey).

Within the unstable area, diverse wetlands highlighted by bright green grass and characterized by hummock-and-hollow surfaces were identified. The distribution of the wetlands area mainly corresponds to the outer limit of the toe bulging portion, clearly evident on the shaded relief and also highlighted by the trend in the contour lines. Figure 8 shows the map of all the morphological features and road damage recognized in the UAV orthoimages of the unstable area.

Based on the observations made by the visual interpretation of the UAV products, we were able to define the boundary of the unstable area. In particular, more than one landslide body has been recognized (Figure 8). Specifically, three main landslide bodies are identifiable and distinctively recognizable in the shaded relief obtained from the DTMs. The smaller one extends between elevations of about 1800 m a.s.l., close to the upstream-side of the S.P. 23, to 1730 m a.s.l., close to the terminal part of the two recognized lateral fissures that distinctively demarcate the landslide boundary. Within this first accumulation body, a minor rotational slide of about 0.43 ha, involving the right side of the S.P. 23 and the retaining wall located at 1786 m a.s.l., is recognizable. By analyzing the distribution of the wetland sectors, which are located in the outer limit of the main bulging portions and testify to permeability variations, the two other landslide bodies were delineated. 

These three landslide bodies are embedded in a more extensive one, of about 9.04 ha with the toe at about 1575 m a.s.l. close to the tributary of the Ripa River. This more extensive phenomenon corresponds to that recognized by the aerial photograms’ analysis, and named first-generation Champlas landslide. Instead, the second-generation one corresponds to the outer limit of the series of embedded rotational slides that occurred in the 2018 reactivation. 

#### 4.2.2. Evidences Derived from RPAS Datasets Comparison

**Figure 9:**
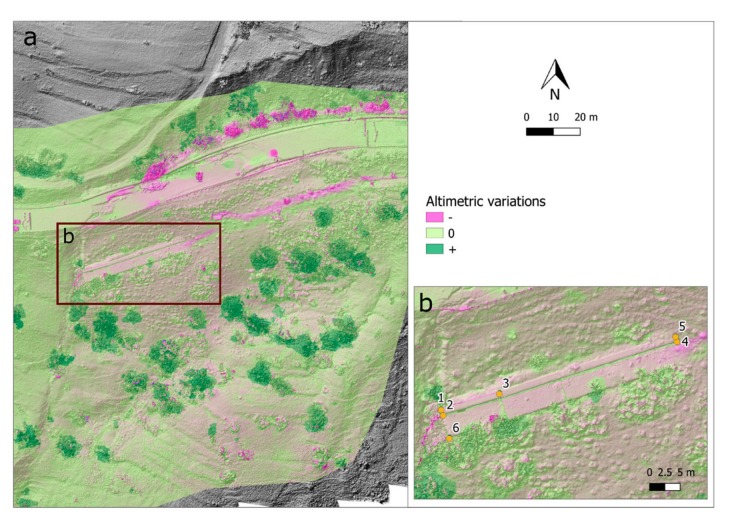
(**a**) Comparison of the altimetric changes from the 15 May 2018 and 14 June 2018 DEMs, illustrating the surface variations in the most damaged area, (**b**) with a focus on the retaining wall, highlighted by the red box, showing the selected points for the planar and height variations computation (see Table 3).

The multi-temporal analysis carried out on RPAS data allowed us to analyze the potential changes that occurred between the 15 May 2018 and the 14 June 2018 flights. It is important to note that both the UAVs flights followed the paroxysmal event and occurred in late April-early May. Therefore, at the time of the flights the main deformation has already occurred. For this reason, by a first visual inspection, we can appreciate that a weak variation occurred between the two UAV acquisition DEMs (Figure 9). The figure illustrates that in about one month, material was removed from the north-western portion of the unstable area, specifically, in correspondence to the pre-existing retaining wall (ENE-WSW oriented) and located in the sector just downstream of the main road. The digging on the road pavement and that for the creation of a runway for the remedial work activity is clearly visible.

We also focused on the pre-existing retaining wall by selecting some well-recognizable features and extracting their three-dimensional coordinates in order to compute both the planar and height variations (Table 3) between the two DEMs.

Six points were selected on the wall. The computed planar dislocation displays a southwest movement, with a displacement ranging from 15 to 25 cm over a period of 30 days. However, considering the variation in altitude a downgrading of about 40–50 cm was detected.

### 4.3. Multispectral Analysis by RGB Camera Mounted on RPAS

**Figure 10:**
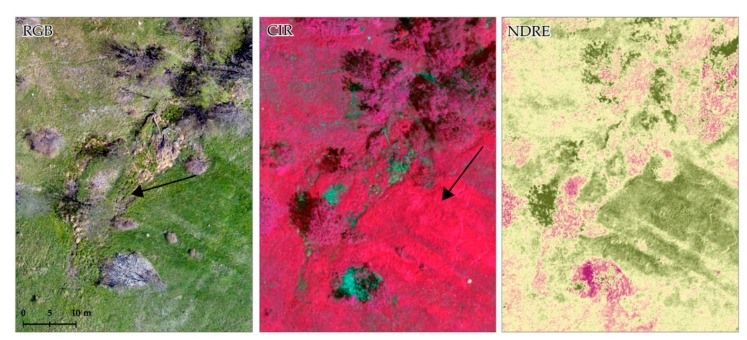
RGB (**left**), CIR (**center**) and NDRE (**right**) of a portion of the investigated landslides revealing hummock-and-hollow pattern (←RGB) and long leaf grass lodging (←CIR) as indicators of water abundance.

The interpretation of multispectral layers (NDRE and CIR maps) and its comparison with RGB data and field surveys revealed a complex pattern of springs and wetlands in the studied area (Figure 10). In particularly, the RGB and CIR composite has highlighted areas featuring hummock-and-hollow texture where, in the field surveys, the presence of springs has been confirmed. Moreover, in the NDRE layer, areas of long leaf grass are emphasized. Their colour suggests their vigor and their pattern indicates the abundance of water and the presence of a surficial flux causing plant lodging.

### 4.4. Terrestrial Images for 3-D Reconstruction

**Figure 11:**
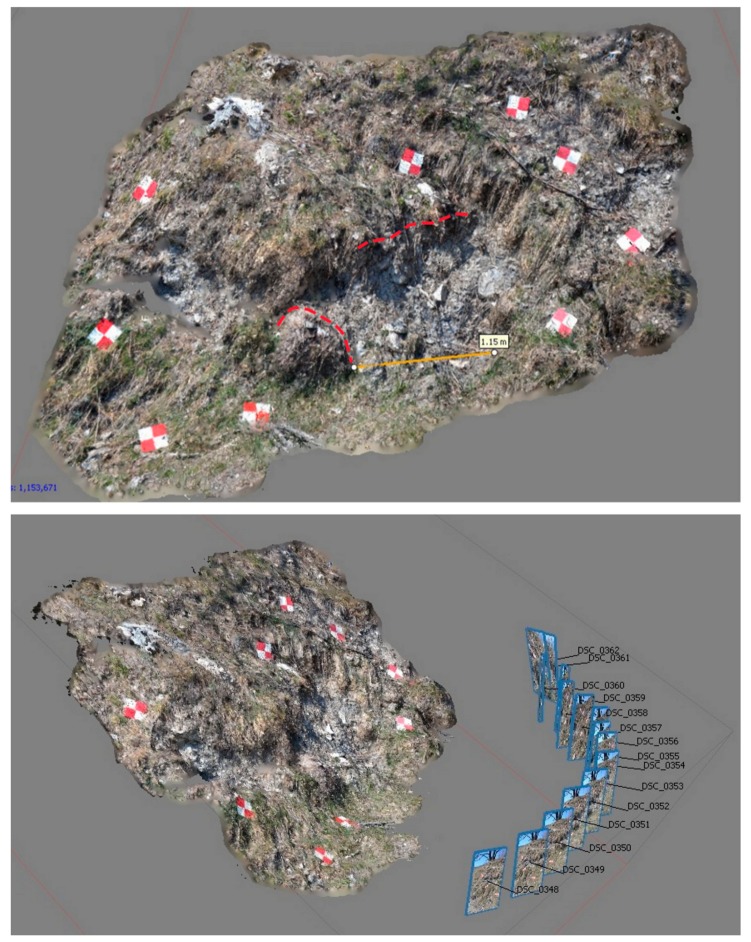
3D-reconstruction of a local portion of the right-side fissure (see Figure 9 to locate the site), processing terrestrial photos (**Up**: manual displacement measure; **down**: positions of image captures).

Local 3-D reconstruction on a sector located along the main lateral scarp on the right side of the studied landslide was generated by taking a sequence of images by smartphone (Figure 11). We processed the acquired images, taking advantage of references of known size (20 × 20 cm) located within the scene. After aligning photos, we used the bench-markers as scale bars for the known distance, in meters, between the targets’ edges. By updating the dataset geometry, the tool refines the scale of the entire model, thus allowing metric measurement.

The lateral fissure in the halfway sector (from 1750 m a.s.l.), is associated to a “fresh” scarp, along which local roto-translational movements are visible, and are materialized in small sods detached from immediately neighboring local scarps. The local displacement was measured in correspondence to a clearly recognizable sod, shown in Figure 11, by measuring the distance between its scarp and the top of the sod. A displacement of about 1.15 m was estimated, mainly due to the dragging effect of the landslide movement.

The displacement computed with this rapid methodology is comparable with the total displacement computed by the RTS, of about 1.3 m recorded during the 2018 reactivation.

### 4.5. Overall Temporal Evolution 

**Figure 12:**
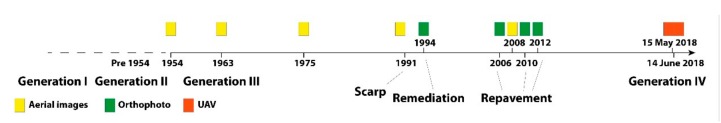
Time line of the Champlas du Col landslide evolution from 1954 to 2018.

The obtained results were combined with the orthophotos available on the *Portale Cartografico Nazionale* web-portal [63] (i.e., 1994, 2006, 2010 and 2012 orthophotos) in order to reconstruct the overall temporal evolution of the area of interest, with a specific focus on the Champlas du Col landslide (Figure 12). A time line created through the combined analysis of the historical aerial photos, the on-line orthophotos and the high-resolution UAV-orthoimages traces the evolution of the area of interest over a 64-year period (from 1954 to 2018), showing the four recognized landslide generations.

**Figure 13:**
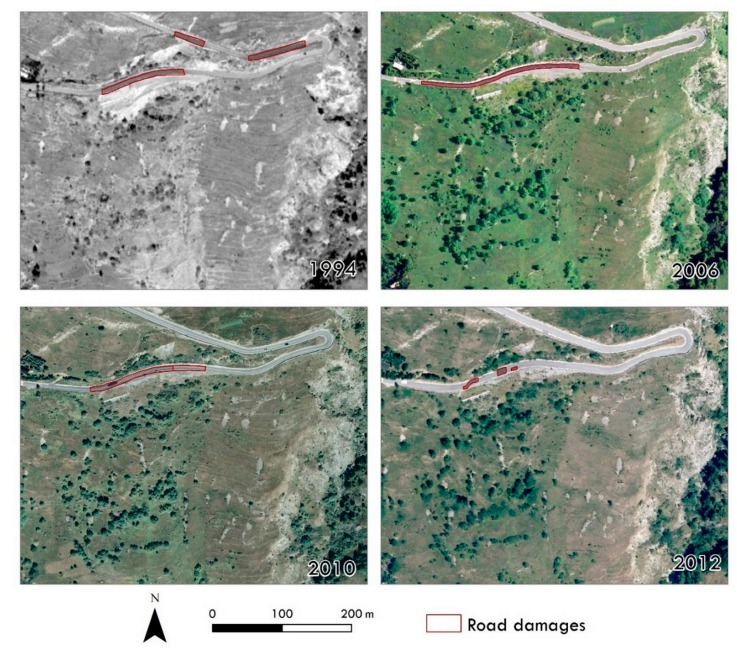
a. Evidence of road repaving visible on the orthoimages available on the “*Portale Cartografico Nazionale*” web-portal [63].

Evidence of road damage, e.g., road repaving (Figure 13) testifies to a long history of instabilities in the Champlas du Col area, which before the construction of the retaining wall involved the entire curvy road (see 1994 orthophoto in Figure 13). Currently, the area involved corresponds to the May 2018 reactivation.

**Figure 14:**
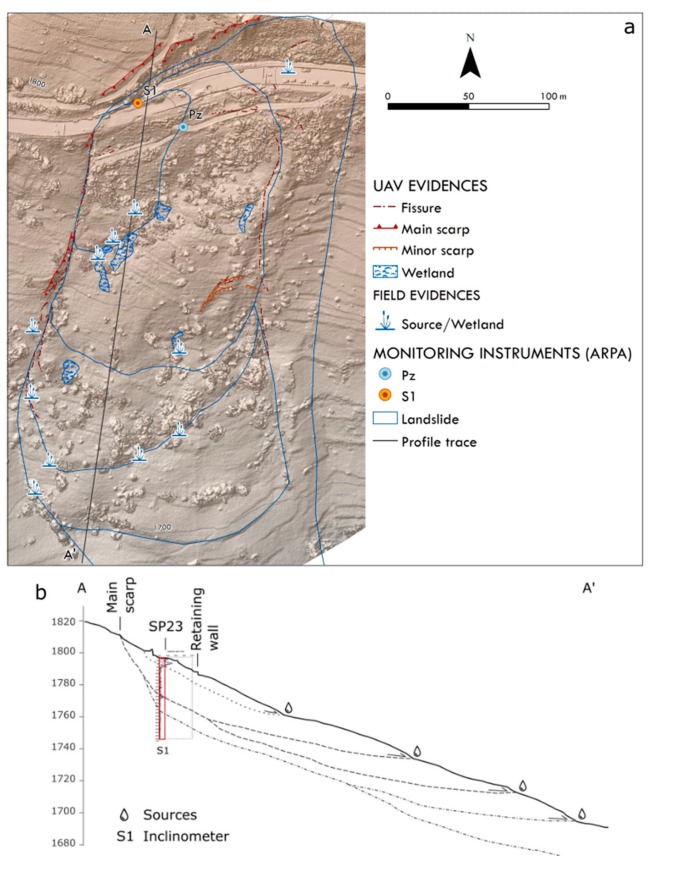
(**a**) Map of the Champlas du Col observed landslides associated with the main morphological features due to the spring 2018 reactivation, and (**b**) longitudinal profile associated with the resulting displacement graph calculated during June–November 2018.

Figure 14 shows the preliminary geomorphological model of the investigated landslide, highlighting the preferential distribution of springs and wetlands in the outer limit of the bulging portions, outlining the series of overlapped landslides, clearly bounded laterally by very open fissures. The existence and the location of many sliding surfaces was confirmed by the inclinometer (S1 in Figure 14) measurements in the period 8 June–13 November 2018. The S1 located one sliding surface at a depth of about 3.5 m, with an initially high speed that gradually decreased almost to zero from early August; a second surface was observed at about 24.5 m, with an initial deformation of about 3.6 cm/year, although this decreased at the beginning of August and settled on values of about 1.4 cm/year.

## 5. Discussion

With regard to the geomorphological framework of the investigated site, the landslide setting is spatially and temporally complex. At the slope scale, the south-facing flank of Mt. Fraiteve is affected by a wide, mass movement known as the Champlas du Col DsGSD, in which a series of minor landslides are set. Thanks to the overall analysis of the final results, we discerned four generations of landslides (Figure 12). The first and the second generation are readily recognizable in the 1954 aerial photograms, and always visible in the following photos. In 1963, a third generation is detectable, located within the body of the second-generation landslide. However, in 1991 photograms, a reactivation of the second-generation landslide was observed and evidenced by a new scarp visible in the sector downstream of the S.P.23. A series of reactivations of the same landslide are recognizable in the orthophotos available on the web. Since the nineties, a series of serious damage to the road network have been observed, as testified by the repaving of the main road surface and the building of the retaining wall just downstream of the S.P.23 national road (Figure 13). After the paroxysmal phenomenon that occurred during the spring of 2018, corresponding to the fourth-generation landslide (see Figure 5), new aerial photos were taken by UAV surveys. The very high-resolution DEMs and orthoimages have led to the recognition and characterization of a series of landslide bodies (Figure 14). The two larger bodies correspond to the oldest phenomena, already recognized in historical reconstructions, within which a series of embedded landslide bodies were recognized on the new UAV images, and which are related to a progressive activation in spring of 2018. In the reactivation, it is important to note that the landslide reactivation starting from late April–early May was a result of the snow melting, which was related to the abundant snowfall during the 2017–2018 winter. The internal data acquired by the Metropolitan City of Turin reported peaks of 5 cm/day from 23 to 30 May 2018 (datasheet 001-76803-00 [56]), with a total displacement of about 1.3 m for the 2018 reactivation. By the 3D-model reconstruction of the terrestrial images taken by smartphone, we could locally estimate the overall landslide deformation (about 1.15 m), which was confirmed by the comparison with the RTS measurements acquired by the MCT. The comparison of the 15 May and 14 June 2018 flights highlights the damage that occurred along the road surface and the remedial work interventions, as well as the pluri-decimeter planimetric and altimetric shift along the retained wall, just downstream of the national road, and which is comparable to the recorded deformations.

This study demonstrates the productive use of the SfM technique applied to multisource images and multi-period acquisition by leveraging historical aerial photograms, high-resolution UAV-images, and terrestrial photos taken by smartphone.

The use of Structure from Motion (SfM) to process aerial and/or terrestrial images constitutes a practical and economic method that is useful for immediate, very high-resolution and detailed mapping in small (0.01–100 km^2^) unstable areas. For landslide hazard analysis, this low-cost photogrammetric method has usually been applied to reconstruct very high-resolution topographic 3D-models, primarily starting from aerial imagery acquired by RPAS [23,25] where the distance of the object is the main factor that influences the accuracy [44]. However, generating DEM and orthoimages by UAV survey associated to flight orientation, based on GCPs measured by GNSS, can generally provide satisfactory results [46,49].

Commonly, multi-source and multi-period DEM/DTM data are a useful tool to characterize and research the mechanism of landslides. In the literature, in comparing the DEM/DTM acquired by diverse sensors and/or instrument at different times, they have been exploited to investigate landslide activity, volume estimation and its variations [71,72,73,74]. It should be noted that by combining information with various precision and resolution methods, collected in different periods, the acquisition scale and the subsequent error estimation of data becomes crucial [49]. In our case, we generated DEMs and orthoimages with highly variable resolution, mainly due to the acquisition scale. The ground resolution of historical aerial photos, ranging from 25 cm/pixel to 1.2 m/pixel is insufficient to be accurately co-registered with the recent UAV dataset (1.65 cm/pixel of resolution). Similar considerations have also been made for the terrestrial images and UAV-images comparison.

As highlighted in previous scientific works [17,18], the availability of historical aerial photograms allows the reconstruction of the previous morphological state, which is useful to the overall temporal evolution assessment of a specific area of interest, and for analyzing the geomorphic framework at regional scale. On the contrary, higher spatial resolution and submeter accuracy of the UAV-images ensures an accurate investigation at local scale, and the topographic information collection [23,44,75].

Theoretically, when the entity of displacement of the gravitational process is particularly remarkable and morphological modifications are highly visible, historical images can be employed in order to reconstruct past geometrical setting on the slope. In these cases, a comparison between diverse sources of data with different resolutions is possible and suitable [76].

The great availability of historical and recent images of the area of interest allowed us to prepare an overall reconstruction of the Champlas du Col landslide. By combining historical aerial photograms, RGB images and multispectral data from UAV surveys, local 3D-reconstruction from terrestrial photos, with field campaign information, and in situ measurement reported by ARPA Piemonte [56], we delineated an initial assessment of the Champlas du Col landslide (Figure 12 and Figure 14).

In our work, the entity of slope change is of the same order of magnitude of the accuracy of aerial photos. For this reason, it is possible to make a direct comparison between old aerial image results and new UAV acquisition. The processing of historical aerial photograms ensured a qualitative analysis of the landscape evolution over a 54-year period (from 1954 to 2008), and allowed the generation of a landslide inventory and the historical reconstruction of the study area. Additionally, the DEMs and orthoimages obtained from the two flights are characterized by adequate resolution for the identification of the main morphological features of the unstable area, as well as of the damage displayed on the road surface of the main road.

## 6. Conclusions

In this paper, a low-cost and repeatable methodology based on SfM to derive valuable information to investigate and characterize active landslides from heterogeneous and multi-scale data sources is presented and discussed. SfM is a valuable tool that is able to compute a 3D point cloud from a sequence of images taken from different points of view. RPAS can be considered an effective solution for high-resolution image acquisition for active landslide analysis. Along with RGB camera equipment, the UAV system can be empowered with other sensors to perform, for instance, multispectral and hyperspectral images. The use of smartphones represents another valid and low-cost approach for capturing terrestrial, RGB images. Likewise, historical aerial images acquired by aerial photogrammetry constitute a relevant information source that can be treated with an SfM approach.

A methodology that combines the use of multiple set of images, acquired from diverse instruments and sensors on various scales has been implemented. This methodology is applied to the Champlas du Col landslide (Sestriere municipality, north-western Italy), for which two UAV surveys were carried out after the spring 2018 reactivation and a series of terrestrial pictures were taken. A multiple set of aerial photogrammetrical images is also available.

The orthophotos obtained from aerial photogrammetric images processed by SfM allowed us to generate a landslide inventory for the area of interest, which was associated to the historical analysis of landslide activity, and is useful to define the evolution of slope instabilities over a 54-year period (from 1954 to 2008). The DEMs and orthoimages obtained from UAV surveys enabled identification of the main geomorphological features due to the recent reactivation and the geological model of the unstable area and characterization of the landslide extension that occurred, in association with the executed field survey and the available in situ data (i.e., inclinometer). Several considerations on the variations that occurred between the two UAV flights products were done for a characterization of the source area up to the area close to the retained wall within the unstable area. In addition, the SfM applied to terrestrial images allowed us to estimate the local deformation rate of the May 2018 re-activation, which resulted in a value comparable to the displacement rate computed by RTS.

Our strategy shows that it is possible to obtain reliable and useful data for landslide hazard definition, employing a cost-effective and rapid methodology based on SfM principles that is easily repeatable to characterize and investigate active landslide in other contexts.

Concerning future improvements, the proposed methodology could be enriched by multi-temporal, multispectral flights in order to monitor the presence and abundance of water in the site. These findings should then be related to the geometrical evolution of the landslide with the purpose of investigating the possible relationship between landslide dynamics and water.

## Figures and Tables

**Table 1:** Aerial photos datasets.

Operator	Year	Number of Images	Dimension [pixel]	Resolution [cm/pixel]	Scale
IGM	1954	3	7269 × 7360	100	1:33.000
Aer-foto	1963	4	4864 × 3898	60	1:18.000
Rossi	1975	4	4864 × 4052	77	1:14.000
Avioriprese	1991	4	7451 × 7977	120	1:38.000
Hansa Lufbild—German Air Survey	2008	9	7680 × 13824	25	1:10.000

**Table 2:** Unmanned aerial vehicle (UAV) survey specifications.

UAV Type	Data Survey	GSD [cm/pixel]	Number of Photograms	Overlap [%]	Sidelap [%]
Sensefly Albris	15/05/18	5	81	80	60
DJI Phantom-4	14/0618	1.65	383	80	60

**Table 3:** Planimetric and height differences computed in correspondence to six points across the retaining wall (see Figure 9) located within the Champlas du Col landslide, between the two DEMs of 15 May 2018 and 14 June 2018, obtained by UAV survey.

Point	Planimetric (cm)	Height (cm)
1	19.8	−39.6
2	16.5	−37.6
3	26.1	−47.3
4	23.0	−50.9
5	19.0	−49.4
6	26.9	−46.6
